# Pancreas-Sparing Duodenal Resection in Colorectal Adenocarcinoma With Local Invasion of the Duodenum: A Case Report

**DOI:** 10.7759/cureus.92840

**Published:** 2025-09-21

**Authors:** Ilango Parthasarathy, Gowtham Karthik V, Suhaildeen Kajamohideen

**Affiliations:** 1 Surgical Oncology, Sri Ramachandra Institute of Higher Education and Research, Chennai, IND

**Keywords:** case report, duodenal cancer, duodenal invasion, locally advanced colorectal cancer, onco-surgery, pancreas-sparing duodenal resection, right colon cancer

## Abstract

A 53-year-old male patient presented with right upper abdominal pain, anemia, and weight loss. Colonoscopy revealed a non-obstructing hepatic flexure growth, and biopsy confirmed moderately differentiated adenocarcinoma. Imaging showed a non-metastatic, locally advanced right colon malignancy involving the ascending colon, hepatic flexure, and proximal transverse colon, with invasion into the pylorus of the stomach and second/third parts of the duodenum, pericolonic nodes, and segmental superior mesenteric vein (SMV) abutment. Following five cycles of neoadjuvant chemotherapy with stable disease on positron emission tomography-computed tomography (PET-CT), the patient underwent an en bloc right hemicolectomy with pancreas-sparing duodenal resection (PSDR). The postoperative course was uneventful, and the patient recovered well. This case highlights PSDR as a feasible alternative to pancreaticoduodenectomy (PD) in select cases of locally advanced right colon cancer (LARCC) invading the duodenum, preserving pancreatic function and reducing morbidity.

## Introduction

Locally advanced colon cancer that adheres to or invades adjacent organs is categorized as T4b disease and requires complete en bloc resection to achieve negative margins, as recommended by surgical oncology guidelines [1]. When the tumor involves the duodenum without infiltration of the pancreas, surgical options include right hemicolectomy with limited duodenal resection and reconstruction [2]. If pancreatic invasion is present, pancreaticoduodenectomy (PD) may be necessary to achieve oncologic clearance [3]. Techniques of pancreas-sparing duodenal resection (PSDR), developed initially for benign and premalignant duodenal conditions, have been adapted in selected malignant cases to preserve the pancreas while maintaining negative margins [4]. In this report, we describe a case of PSDR combined with right hemicolectomy for locally advanced right colon adenocarcinoma with duodenal invasion, and review the relevant literature.

## Case presentation

A 53-year-old male presented with right upper abdominal pain for three months, associated with anorexia, early satiety, and progressive weight loss of 6 kg. He also reported fatigue and episodes of melena for the past month, but denied hematemesis, jaundice, or altered bowel habits. There was no history of previous abdominal surgery. Family history was negative for colorectal or gastrointestinal malignancies. He was a non-smoker and did not consume alcohol.

On examination, the patient appeared pale with conjunctival pallor but was hemodynamically stable. Abdominal examination revealed mild tenderness in the right upper quadrant without palpable mass, hepatomegaly, or ascites. Per rectal examination revealed altered black stools, but no intraluminal growth.

Laboratory evaluation revealed hemoglobin of 8.9 g/dL, mean corpuscular volume of 72 fL, and serum ferritin of 10 ng/mL, consistent with iron deficiency anemia. Peripheral smear showed microcytic hypochromic cells. Stool occult blood was positive. Liver and renal function tests were within normal limits. Serum carcinoembryonic antigen (CEA) was 4.48 ng/mL.

**Figure 1:**
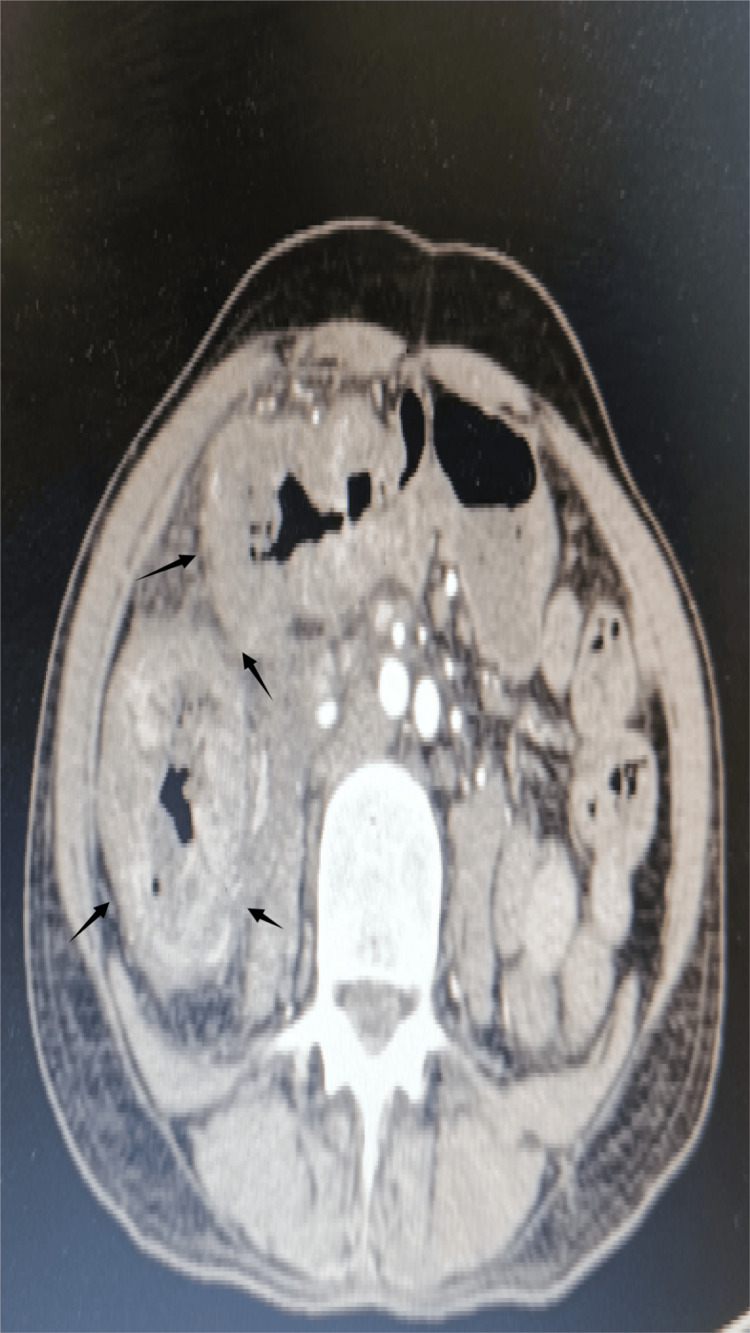
PET-CT demonstrated a locally advanced right colon malignancy with invasion into the pylorus and duodenum PET-CT: positron emission tomography-computed tomography

**Figure 2:**
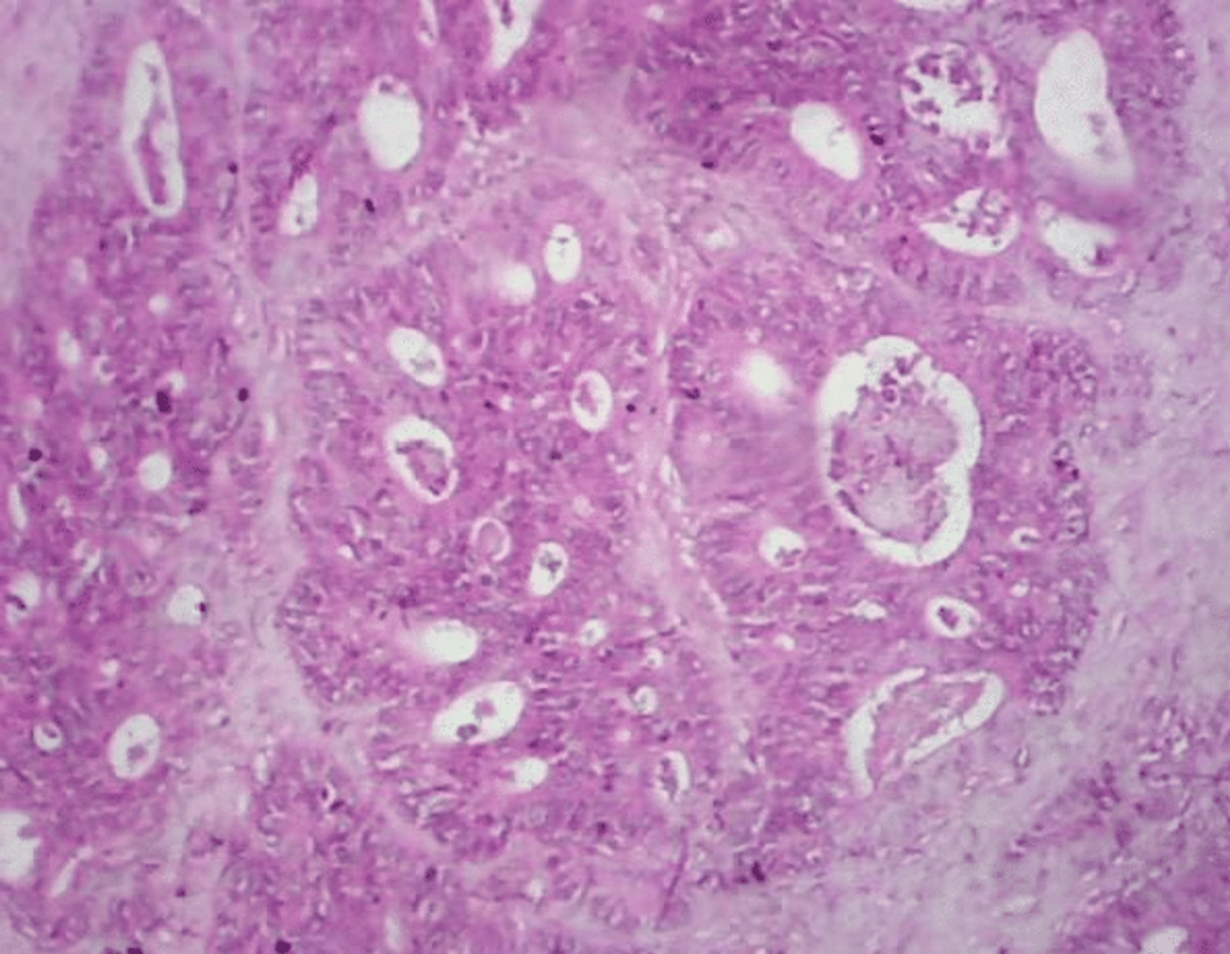
Histopathological examination of the resected specimen showing moderately differentiated adenocarcinoma infiltrating the duodenal wall The tumor exhibits irregular glandular structures with nuclear atypia and desmoplastic stromal reaction (H&E stain, ×100).

PET-CT demonstrated a locally advanced right colon malignancy with invasion into the pylorus and second part of the duodenum, but no distant metastases (Figure 1). Upper gastrointestinal endoscopy showed normal mucosa up to the duodenum without intraluminal growth. Colonoscopy revealed a non-obstructing hepatic flexure mass, and biopsy confirmed moderately differentiated adenocarcinoma (Figure 2).

The patient received five cycles of CAPEOX chemotherapy (capecitabine 1000 mg/m² orally twice daily on days one to 14 and oxaliplatin 130 mg/m² intravenously on day one of each 21-day cycle), with stable disease on interval imaging. Recurrent anemia during chemotherapy was managed with blood transfusions and oral hematinics. ECOG performance status remained grade 1 throughout.

Intraoperatively, the tumor was seen invading the second portion of the duodenum without evidence of pancreatic head infiltration. Considering these findings, a pancreas-sparing approach was planned. En bloc resection of the involved transverse colon segment was performed along with wedge resection of the duodenal wall. Frozen section confirmed negative margins. The duodenal defect was closed primarily in two layers, and bowel continuity was restored. No pancreatic or biliary resection was required. A preoperative ERCP-guided pancreatic duct stent was placed for intraoperative guidance.

**Figure 3:**
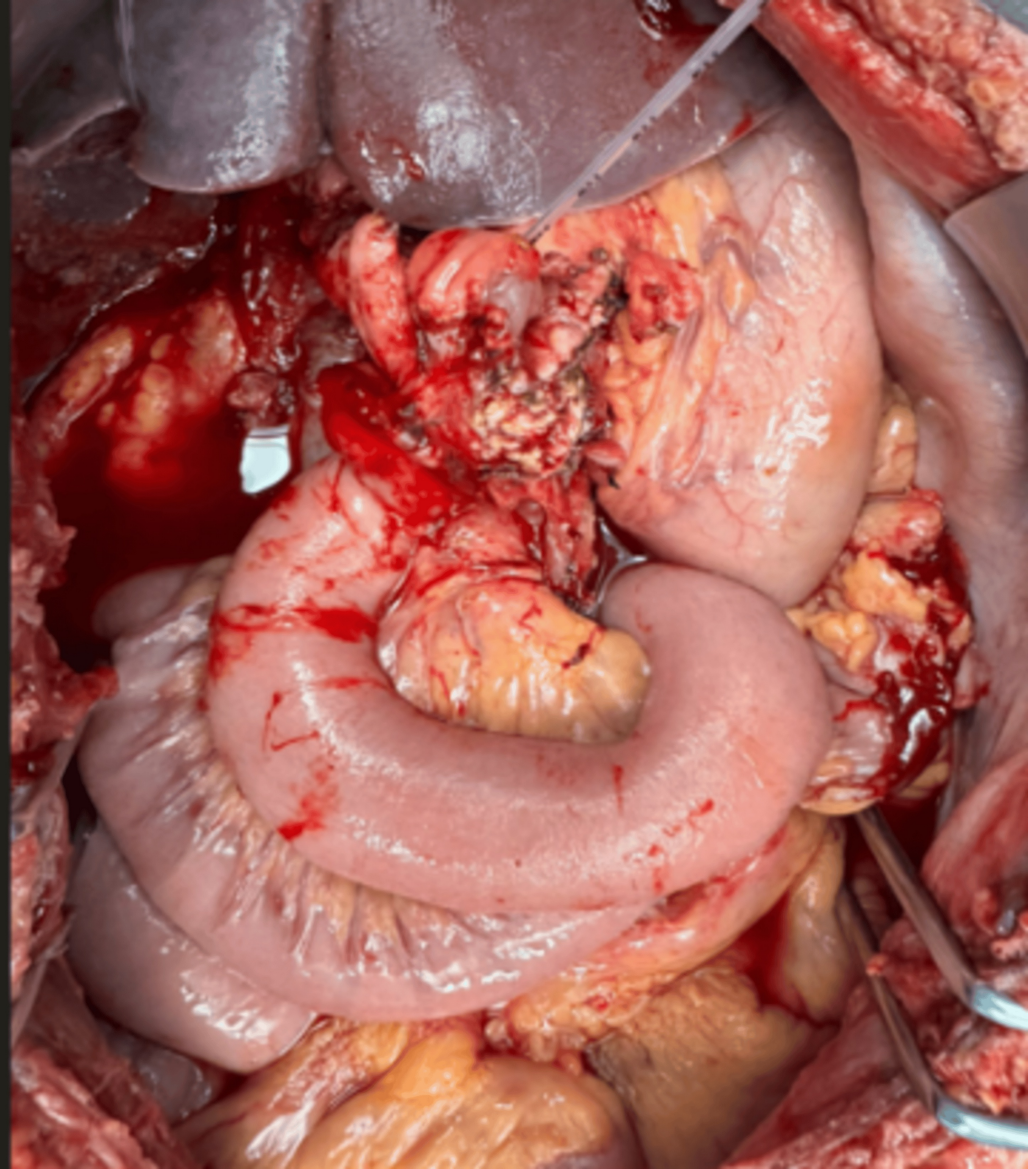
Intraoperative image showing hepatic flexure tumor with invasion into the duodenum Hepatic flexure tumor of the colon with direct invasion into the duodenum. Surrounding small bowel loops and mesentery are visible. The lesion is exposed during surgical exploration, prior to resection.

Laparotomy revealed a hepatic flexure growth invading the pylorus and third part of the duodenum, with a nodal mass adherent to the superior mesenteric vein (SMV) (Figure 3). Careful exploration revealed no invasion of the pancreas, ampulla, or major vessels beyond the nodal mass. A right hemicolectomy with D2 lymphadenectomy was performed, combined with pyloroduodenal resection. The duodenum was dissected to preserve pancreatic head vascularity from the superior mesenteric artery (SMA) and gastroduodenal artery (GDA). The periampullary wall was uninvolved; a 1 cm duodenal margin was confirmed intraoperatively by frozen section. The involved SMV segment was resected after confirming adequate venous drainage via the first jejunal tributary. Gastrointestinal continuity was restored with a common jejunal loop: proximal end-to-side anastomosis to the duodenal ampullary mucosa (guided by the stent) and distal side-to-side gastrojejunostomy.

**Figure 4:**
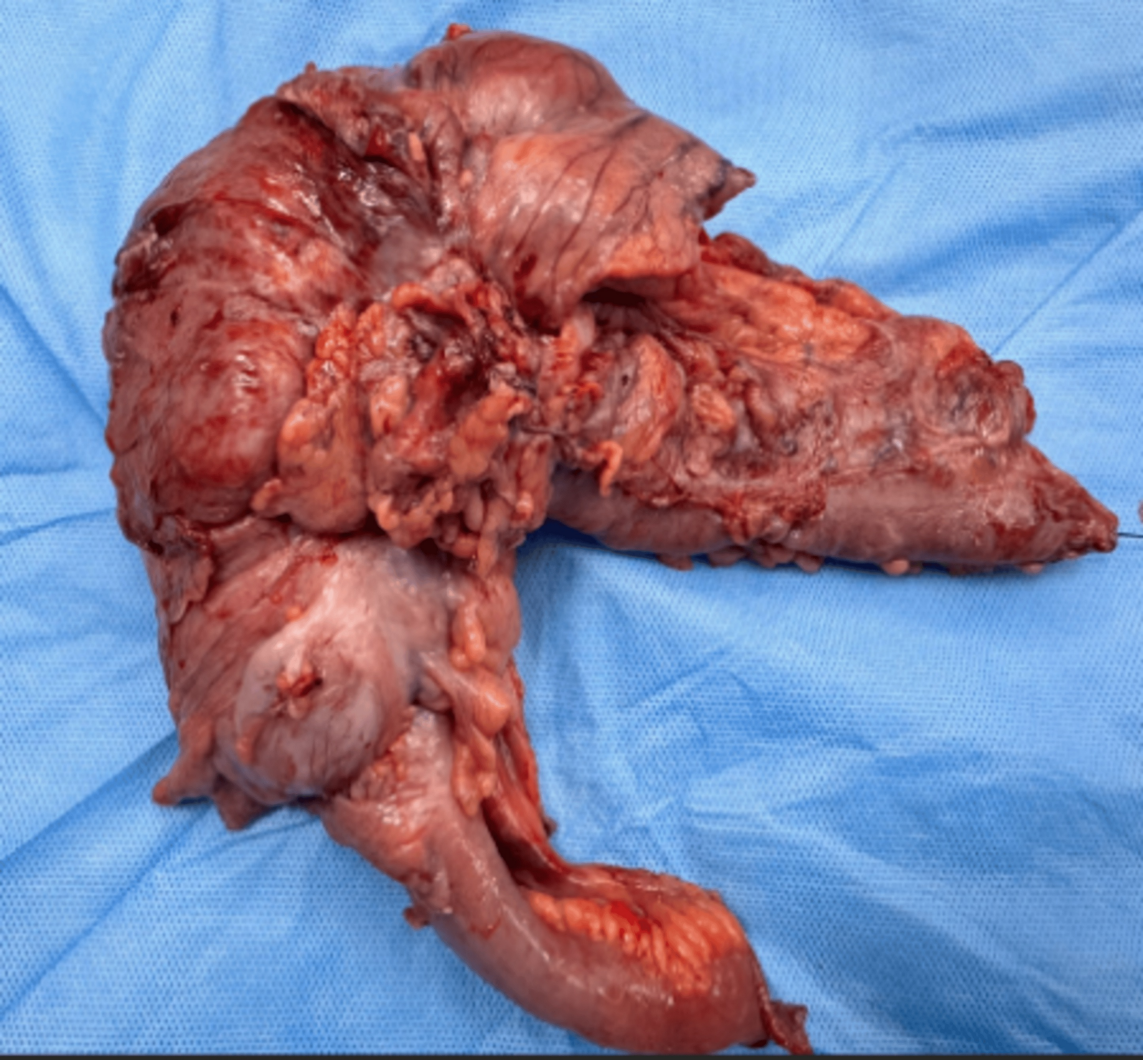
Image of the resected specimen with the involved duodenum

The resected specimen included the hepatic flexure, pylorus, and involved duodenum (Figure 4). Final pathology confirmed moderately differentiated adenocarcinoma infiltrating the duodenal wall without pancreatic or ampullary involvement. R0 resection was achieved with negative duodenal and radial margins (D0, R0 status).

Postoperative course

The patient’s immediate postoperative course was documented in SOAP format. Subjective: The patient reported mild abdominal pain with a VAS score of 4/10 on postoperative day (POD) 1, which decreased to 2/10 by POD3. Objective: The patient remained afebrile and hemodynamically stable. Drain output was initially serosanguinous, later becoming bilious. Bowel sounds were present, flatus was passed on POD3, and ambulation was achieved on POD2. Assessment: A grade A pancreatic leak was suspected on POD4 due to bilious drain output with drain fluid amylase levels three times higher than the serum value. Plan: The leak was managed conservatively with continued drainage, octreotide administration, bowel rest, and gradual reintroduction of oral feeding. By POD10, the drain output had resolved, and the drain was removed.

The patient was discharged on POD12, tolerating diet and mobilizing independently. Follow-up at one month and three months showed satisfactory recovery with no evidence of recurrence on imaging. The patient is currently on adjuvant CAPEOX chemotherapy, planned for three additional cycles.

## Discussion

Direct invasion of adjacent organs occurs in 5%-11% of advanced right-sided colon cancers, with the duodenum being more commonly involved than the pancreas [5]. Such presentations are rare but clinically significant because they pose unique diagnostic and surgical challenges. Early recognition and appropriate surgical planning are essential to achieve oncologic clearance while minimizing morbidity.

En bloc resection is considered the gold standard for T4b colon cancers, as it maximizes the likelihood of achieving an R0 resection and thereby improves long-term survival [6]. When pancreatic parenchymal invasion is confirmed, PD is generally recommended. However, PD carries considerable morbidity and mortality, with reported complication rates of 40%-50% and perioperative mortality up to 5%-10% in some series [7].

In carefully selected patients without pancreatic invasion, PSDR has been reported as a feasible alternative [8]. Techniques include limited duodenal resection with primary closure for small defects, or reconstruction using duodenojejunostomy or jejunal patching for larger defects [9]. A systematic review demonstrated that PSDR can provide acceptable oncologic outcomes while reducing perioperative morbidity compared with PD [10]. Case series further support this, showing low leak rates, rapid recovery, and consistent achievement of negative margins when reconstruction is tailored to the size and location of the defect [9,11].

Our patient’s experience aligns with these findings. Technical challenges included: achieving a 1 cm margin at the periampullary region, preservation of pancreatic head vascularity via the SMA and GDA, and SMV segment resection with confirmation of venous drainage through the first jejunal tributary. Intraoperative frozen section confirmed negative margins. Postoperatively, the patient developed a grade A pancreatic leak, which was successfully managed conservatively with octreotide, drainage, and gradual resumption of oral intake.

This case highlights the balance between oncologic radicality and operative morbidity. PSDR allowed en bloc clearance of the tumor and involved duodenal wall while avoiding the high morbidity of PD. The patient’s postoperative recovery was uneventful aside from the conservatively managed pancreatic leak. Accumulating evidence suggests that PSDR, though technically demanding, is a safe and effective option in patients with duodenal wall invasion without pancreatic parenchymal involvement [8-11].

## Conclusions

PSDR represents a valuable surgical option in select cases of right colon cancer with direct duodenal invasion in which the pancreas and ampulla are uninvolved. By avoiding PD, PSDR reduces morbidity while achieving oncological clearance. Our case demonstrates technical feasibility and uneventful recovery, reinforcing PSDR as an important alternative in complex colorectal malignancies.
